# Supplementary material for: Synthesis and Antibacterial Activity of Cationic Amino Acid-Conjugated Dendrimers Loaded with a Mixture of Two Triterpenoid Acids
Source: Polymers (Basel). 2021 Feb 9;13(4):521. doi: 10.3390/polym13040521 (PMC7916190; doi:10.3390/polym13040521)
Supplement: Supplementary file 1 [file polymers-13-00521-s001.pdf]

polymers

MDPI

Supplementary Information

Synthesis and Antibacterial Activity of Cationic Amino Acids-Conjugated Dendrimers Loaded With a Mixture of Two Triterpenoid Acids

Anna Maria Schito [1], Gian Carlo Schito [1] and Silvana Alfei [2,*]

[1] Department of Surgical Sciences and Integrated Diagnostics (DISC), University of Genoa, Viale Benedetto XV, 6, I-16132 Genova, Italy, amschito@unige.it (A.M.S); giancarlo.schito@unige.it (G.C.Z.)
[2] Department of Pharmacy (DiFAR), University of Genoa, Viale Cembrano 4, I-16148, Genova, Italy, alfei@difar.unige.it (S.A.)
* Correspondence: alfei@difar.unige.it; Tel.: +39-010-335-2296 (S.A.)

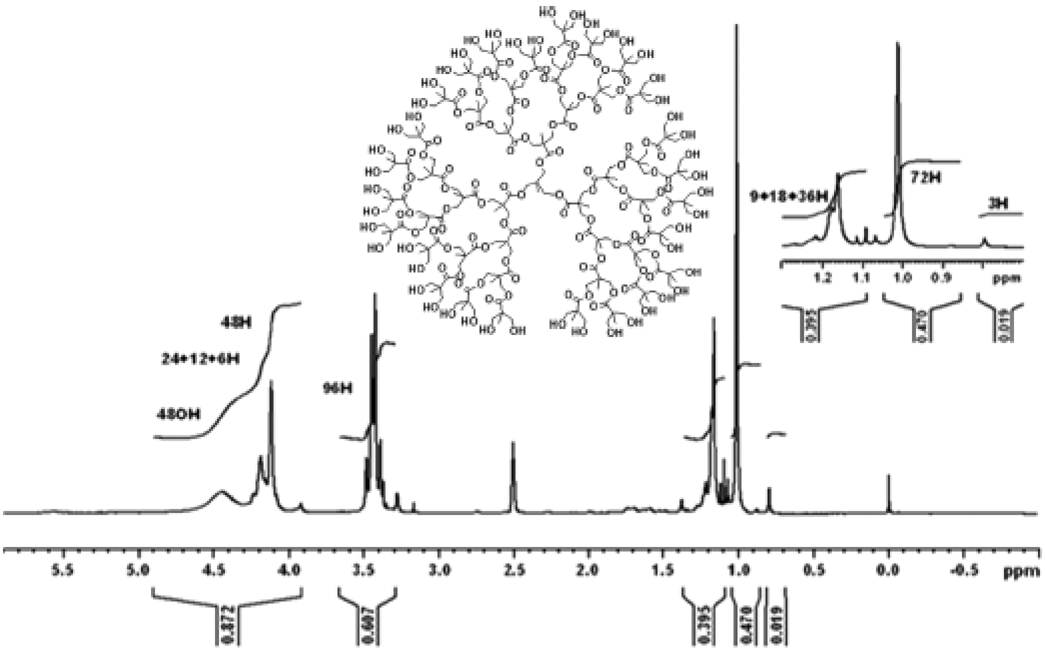

Figure S1. ^{1}H NMR of G4 (300 MHz, DMSO-*d6*).

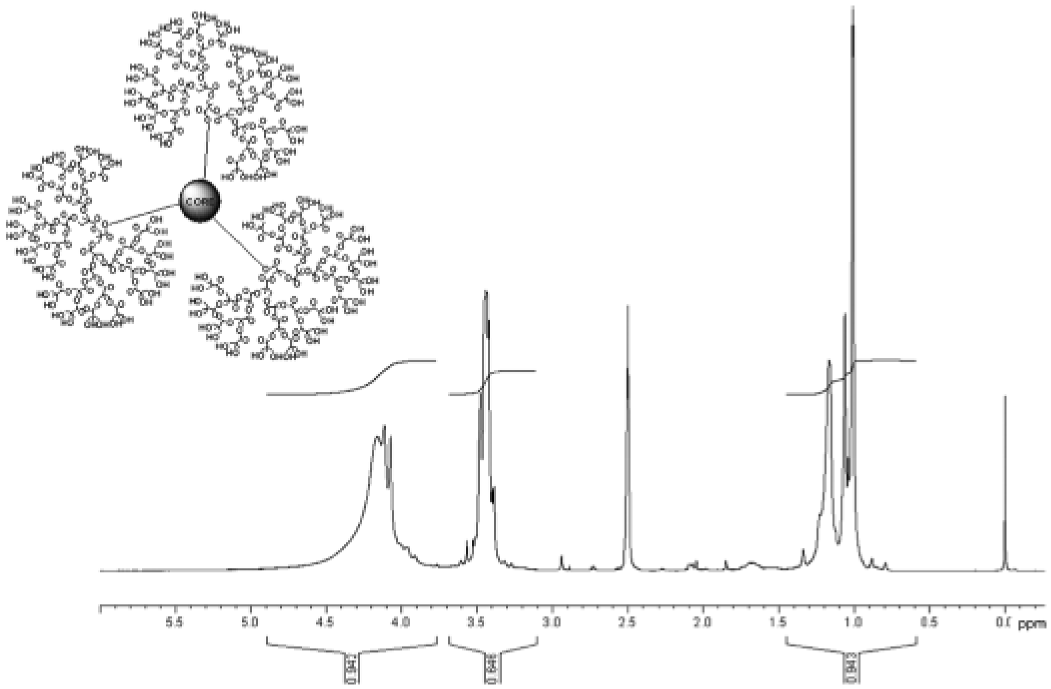

Figure S2. ^{1}H NMR of G5 (300 MHz, DMSO-*d6*).

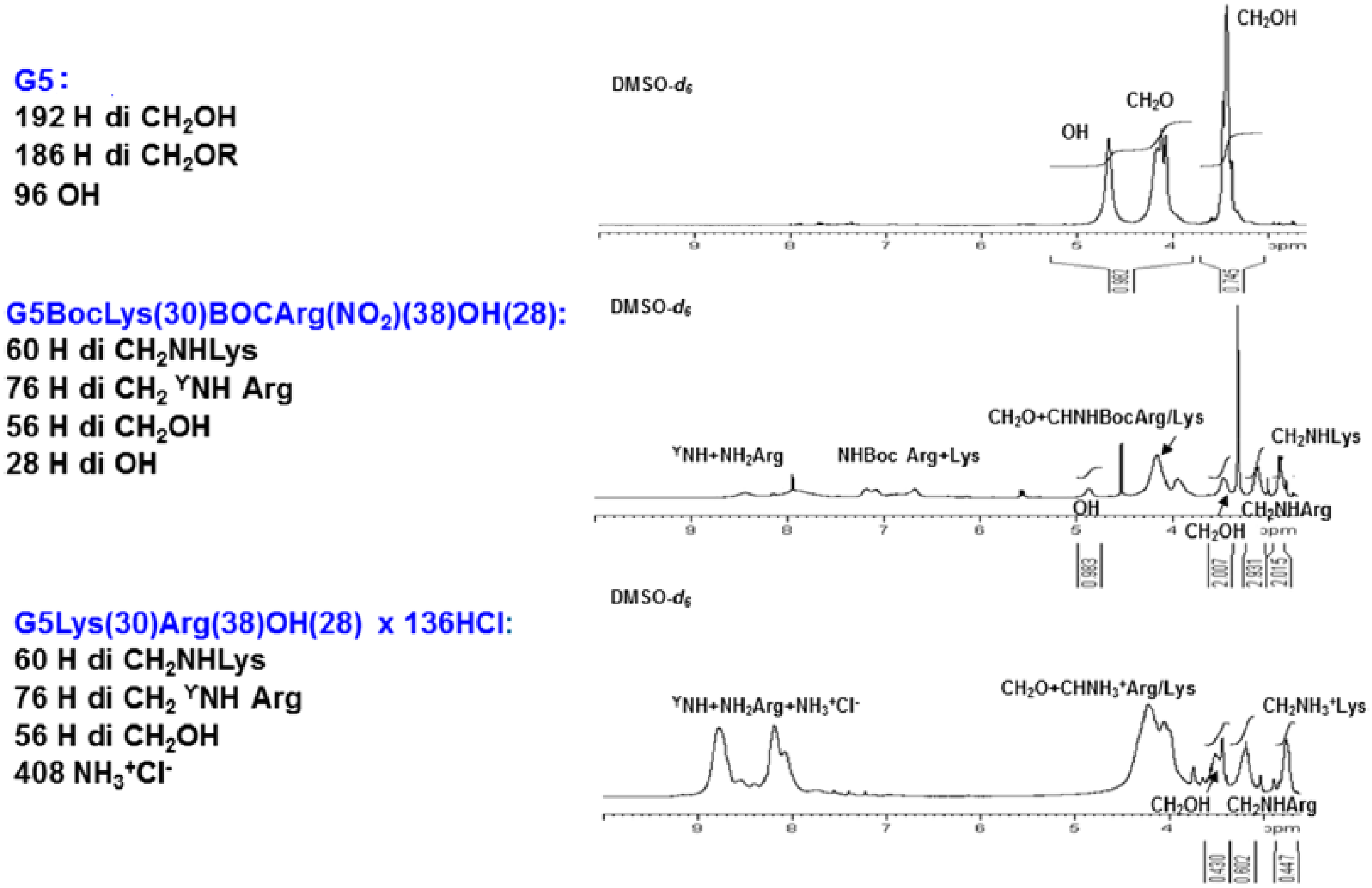

Figure S3. ^{1}H NMR of G5, Boc-protected intermediate and the final cationic dendrimer G5R(38)K(30)OH(28) (300 MHz, DMSO-*d6*).

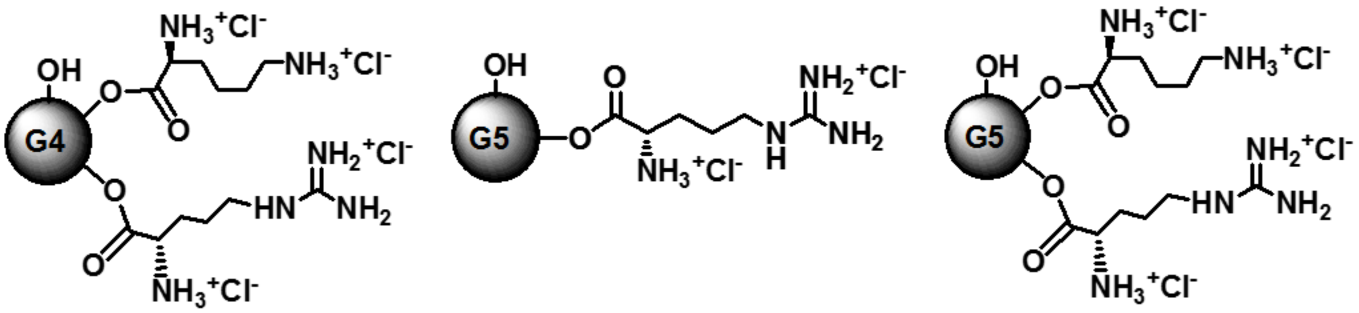

Figure S4. Starting from the left side, simplified structures of G4R(16)K(19)OH(13), G5R(66)OH(30) and G5R(38)K(30)OH(28).

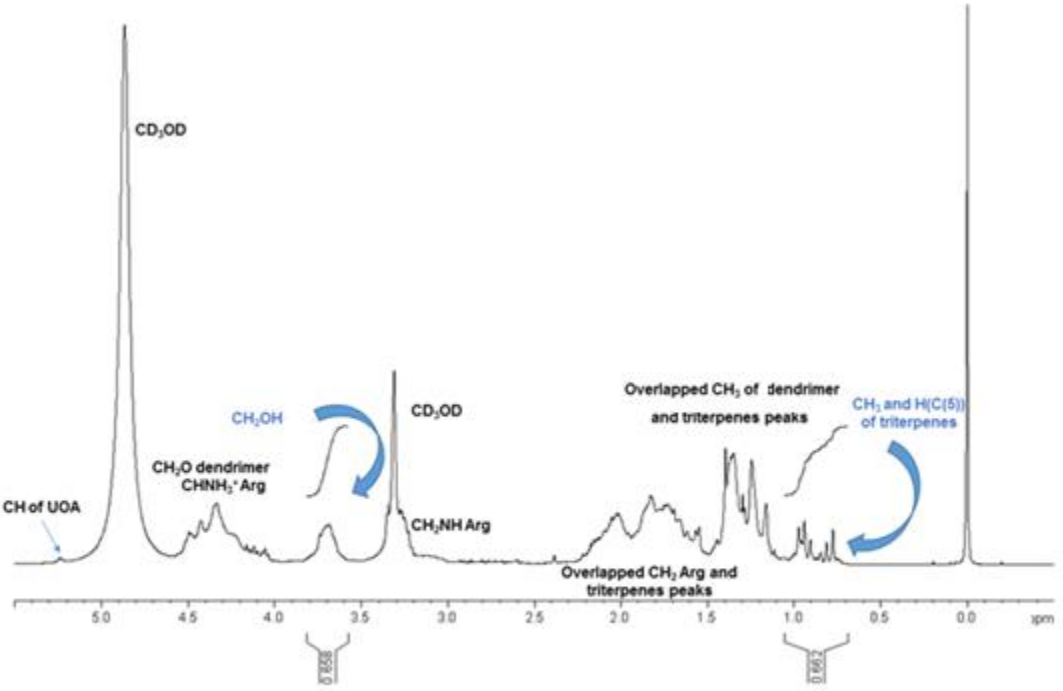

Figure S5. ^{1}H NMR spectrum of G5R(66)UOA(3) in CD_3OD (300MHz).

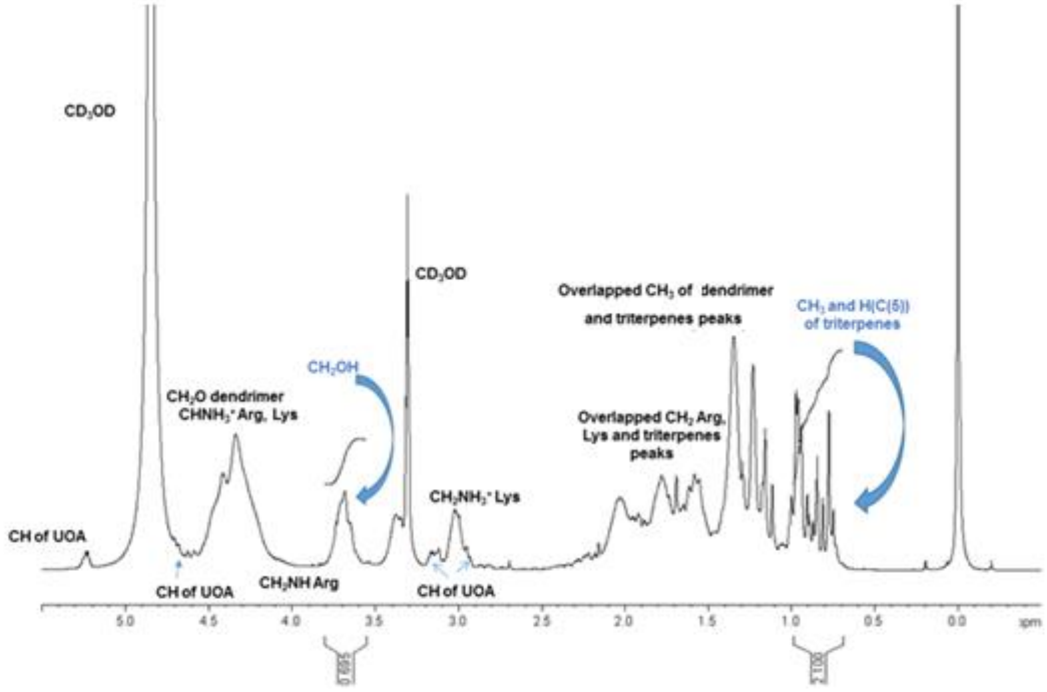

Figure S6. ^{1}H NMR spectrum of G5R(38)K(30)UOA(8) in CD_3OD (300MHz).

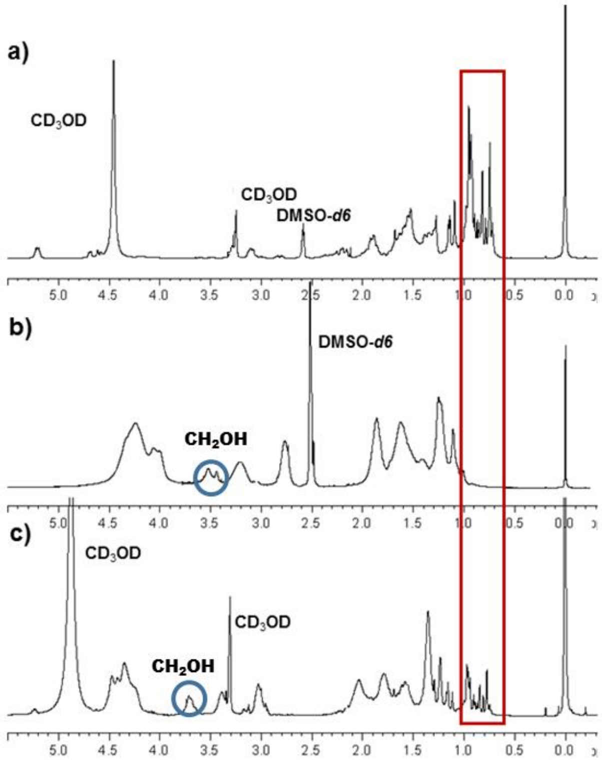

Figure S7. Comparison between the 1H NMR spectra of UOA (a), the cationic empty dendrimer G4R(16)K(19)OH(13) and the UOACD G4R(16)K(19)UOA(4). In the unloaded dendrimer, the signal of the CH_2OH group appears slightly shifted due to the different solvents used during spectra acquisition.

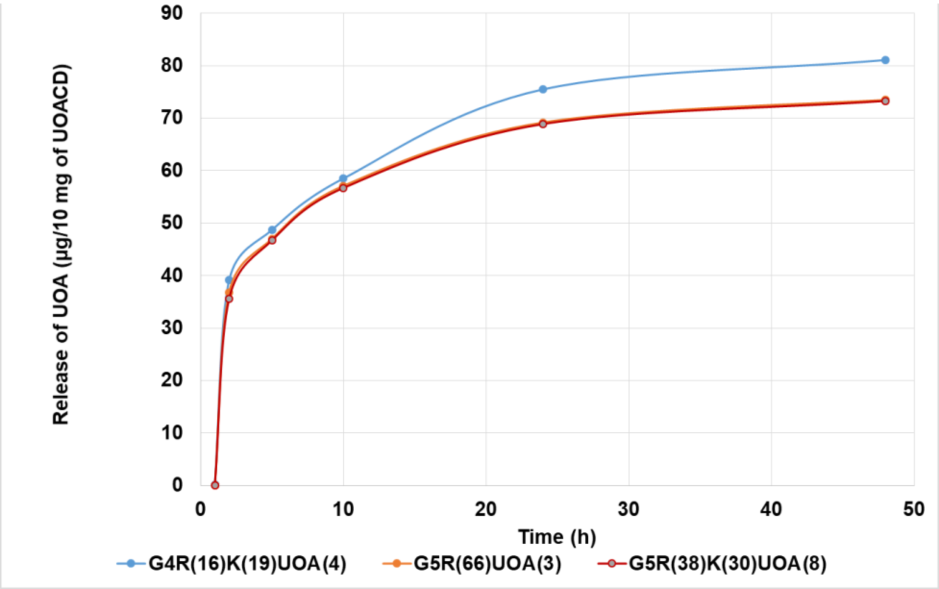

Figure S8. Release profile of UOA from UOACDs.
